# Case of Gun-shot Wound of the Face

**Published:** 1842-09

**Authors:** J. F. Peebles

**Affiliations:** Petersburg


*Case of Gun shot Wound of the Face, with loss of a greater proportion of the Tongue, and extensive lesion of the bony structure, successfully treated; together with an account of interesting nervous Phenomena, resulting from the injury.* By J. F. Peebles, M. D.—In the month of August, 1840, Washington Perkins, a middle aged man of robust constitution, but intemperate in his habits, induced by a fit of jealousy to attempt self-destruction, placed the muzzle of a fowling-piece, charged with duck-shot, immediately below and in front of the angle of his right jaw, and discharged the gun with his foot.

I saw him a few minutes after the accident; the hemorrhage was frightful, though he was composed and sitting up in bed. Upon examination I found an entire breach in the inferior maxillary bone at the point where the shot had been received of more than inch in length, involving the loss of the two lower molar teeth. Passing obliquely upwards through the mouth, the tongue was torn across in the line of the shot, all the free portion of it with the attaching frænum, completely severed and thrown forward between the front teeth. The charge passed out through the antrum about three quarters of an inch below the eye, carrying with it also the two cuspidati and their alveolar processes.

In addition to the injury of the inferior maxillary bone already named, there was a transverse fracture at the symphysis. Owing to the nature and peculiar situation of the injury, the means for arresting the profuse hemorrhage were confined principally to rest in the recumbent posture and cold applications to the head, face and neck. But the quantity of blood which had found its way into the stomach, and still continued to trickle down the throat, despite our efforts to prevent it, very much embarrassed and impeded their effects by the frequent retchings it induced. As soon as fainting came on, however, firm coagula formed in the cavity of the mouth and the orifices of the wounds, and the hemorrhage entirely ceased. In this state he was left for the night with directions for the *diligent* continuance of rest and the cold applications to the head and face.

During the night, from the frequent gratification of his intense thirst, the coagula were removed, and the bleeding partially returned, but a bit of ice in the mouth controlled it until the following morning, when all oozing was promptly suppressed by pledgets of lint soaked in a solution of kreosote applied on the bleeding surface.

His face and wounds now presented the following appearance. His mouth, particularly the lower portion of it, was drawn to the left side; he complained of feeling a notch in the glass from which he drank, owing to loss of sensation in the right portion of his lower lip, phenomena which indicated lesion in the motor and sensitive nerves which supply the lower portion of the face. Over the inferior maxillary bone where the charge had entered there was a circular but jagged wound of over an inch in circumference, either way, extending up on the neck. On introducing the finger the fractured ends of the inferior maxillary bone were found to present a remarkable peculiarity. Instead of being shattered, and split or splintered as might have been expected from the violence of the accident and the nature of their structure, the ends of the bone were found presenting regular and transverse surfaces, as if only that plug of bone had been clearly removed which had received the violent charge, without material injury to the adjoining portion. The osseous system of this man had always exhibited evidence of remarkable fragility. He had suffered fracture of the thighs five different times, and as it is usual in individuals suffering from fragilitas ossium, the bones had in each instance united, with but little inconvenience, and with remarkable facility. Was not the regularity and favorable nature of the fracture to be attributed to this condition of the osseous system, which, doubtless, was general?

The end of the tongue was retracted and swollen, so as considerably to impede deglutition. The left antrum was exposed, while the external wound above presented the form of a triangular incision, with a flap perfectly preserved and thrown back. This was now brought down, the parts adjusting themselves perfectly together, and confined with adhesive plaster.

The portion of inferior maxillary bone between the symphysis and the breach at the angle had fallen inwards, protruding the teeth longitudinally into the mouth, and was so loose and detached as to occasion some thoughts of the propriety of its immediate removal. It was however erected into its proper position and confined as securely as the circumstances would permit, and for the few succeeding days the patient remained tolerably comfortable. After this time for the succeeding ten days alarming hemorrhage from time to time continued to recur, but by the diligent continuance of cold applications to the head and face, together with the topical application of kreosote to the bleeding surface of the tongue and cheeks, it was checked; when he ceased to suffer further annoyance or danger from this score. At the end of the second week the external wound over the antrum had healed by the first intention, and although the antrum still remained exposed, the wound in the superior maxillary bone gave no further inconvenience. The tongue was also well, and healthy granulations were shooting up in the wound in the right cheek. The detached piece of inferior maxillary bone became now the most embarrassing feature of the case. As it was not fastened at either end it was impossible, such was the state of the external wound, now to adopt any means by which it could steadily be held in its proper place a sufficient length of time for union to occur; and for upwards of a month the prospect of its removal, either by necrosis or excision, was improbable. About the middle of the second month, however, it had united at the symphasis, and at once the health of the patient, which had been kept feeble by the exhausting discharge and irritation about the mouth, rapidly improved.

From this time the contraction of the muscles gradually approximated the ends of the inferior maxillary bones, and at the termination of the fourth or fifth month they became united, and the cure was complete.

The following is the condition of the man's face at this time. Considering the great loss of bone, its general contour is but little altered. The inferior maxillary bone, though somewhat shortened, and with the exception of a slight protrusion where the union at the symphasis occurred, is perfect and sufficiently strong for the purposes of mastication. The remaining portion of the tongue, doubtless from the long inactivity of its muscles, is atrophied to a mere membrane. It has well developed papillæ, however, and the taste still remains unimpaired. It affords no assistance in speech, or in mastication, (the finger being used in the latter operation to keep the material between the teeth,) but remains motionless on the floor of the mouth. The deglutition is perfect. The membrane covering the exposed antrum is healthy. The mouth is still partially drawn to the left side, and the muscles of the lower part of the right side of the face take no part in the expression of the countenance. During laughter they are motionless, grotesquely distorting the face. But as this state of things is confined to the lower part of the face, the eyelids and muscles on the side of the nose remaining unaffected, it is probable that the lesion is confined to the lowest branch of the *portio dura* on the face. A portion of the motor branch of the fifth pair is also complicated in the lesion, as the muscles of the right cheek are atrophied, and flap, from a want of consent between their action and the motions of the jaw, in such a manner between the teeth as to prevent mastication on that side. But the *sensation* has returned in the lip. This occurred soon after union had begun at the fracture near the symphysis, proving consequently, that the paralysis which had occasioned the loss of sensation was owing only to the pressure exerted by the displacement of bone, on the branch of the third division of the fifth pair of nerves, which emerges from the anterior mental foramen to be distributed on the lip. This man has returned to his old habits, and is frequently seen intoxicated about the streets, yet the bones of the face and the mucous membrane of the mouth continue perfectly healthy. Indeed the whole history of the case manifests a hardihood, and a strong disposition to the healthy reparation of injury in the osseous system which, I think, is quite remarkable.

PETERSBURG, March 22, 1842.

